# CD44 variant inhibits insulin secretion in pancreatic β cells by attenuating LAT1-mediated amino acid uptake

**DOI:** 10.1038/s41598-018-20973-2

**Published:** 2018-02-12

**Authors:** Nana Kobayashi, Shogo Okazaki, Oltea Sampetrean, Junichiro Irie, Hiroshi Itoh, Hideyuki Saya

**Affiliations:** 1 Division of Gene Regulation, Institute for Advanced Medical Research, Keio University School of Medicine, Tokyo, 160-8582 Japan; 2 Division of Endocrinology, Metabolism, and Nephrology, Department of Internal Medicine, Keio University School of Medicine, Tokyo, 160-8582 Japan

## Abstract

CD44 variant (CD44v) contributes to cancer stemness by stabilizing the xCT subunit of system xc(−) and thereby promoting its glutamate-cystine antiporter activity. CD44 has also been implicated in autoimmune insulitis and inflammation in diabetic islets, but whether CD44v regulates insulin secretion has remained unclear. Here we show that CD44v inhibits insulin secretion by attenuating amino acid transport mediated by the L-type amino acid transporter LAT1. CD44v expression level was inversely related to insulin content in islets of normal and diabetic model mice. Knockdown of CD44 increased insulin secretion, the intracellular insulin level, and the transport of neutral amino acids mediated by LAT1 in Min6 cells. Attenuation of the uptake of neutral amino acids with a LAT inhibitor reduced insulin secretion and insulin content in Min6 cells, whereas overexpression of LAT1 increased insulin secretion. Moreover, inhibition of LAT1 prevented the increase in insulin secretion and content induced by CD44 depletion in Min6 cells. Our results thus implicate CD44v in the regulation of insulin secretion and reveal that amino acid transport is rate limiting for such secretion. They further suggest that amino acid transport mediated by LAT1 is a potential therapeutic target for diabetes.

## Introduction

CD44 is an adhesion molecule expressed at the cell surface and is thought to be a marker of cancer stem cells^1–3^. The CD44 gene in humans contains 20 exons and generates multiple protein isoforms, including both standard (CD44s) and variant (CD44v) isoforms, through alternative transcript splicing^4,5^. CD44v is expressed in various types of cancer and appears to be related to tumor progression and metastasis^5,6^. We recently showed that CD44v interacts with and thereby stabilizes the xCT subunit of system xc(−), a glutamate-cystine transporter expressed at the cell surface, and that the resulting increase in cystine import gives rise to an increase in the intracellular level of reduced glutathione^7,8^. CD44v thus protects cancer cells from oxidative stress and thereby promotes tumor growth and survival^9^. The function of CD44v in normal tissues has remained unknown, however.

Pancreatic β cells contribute to maintenance of glucose homeostasis by sensing the surrounding glucose level and adjusting their rate of insulin secretion accordingly. Regions of human pancreatic islets affected by autoimmune attack in type 1 diabetes were found to overlap with areas expressing the CD44 ligand hyaluronan^10,11^. Furthermore, islets of diabetic mice with autoimmune insulitis were shown to express CD44, and administration of a monoclonal antibody to CD44 ameliorated the insulin-dependent diabetes of these animals^12^. In addition, CD44-deficient mice with obesity manifest enhanced glucose tolerance compared with wild-type obese mice due to suppression of inflammation in adipose tissue^13^. These findings implicate CD44 in the regulation of insulin secretion or insulin sensitivity.

LAT1-4F2hc is a heterodimeric L-type amino acid transporter that is expressed in various tissues^14–18^ and is thought to mediate in particular the transport of amino acids into highly proliferative cells^19^. LAT1 transports isoleucine, histidine, leucine, methionine, phenylalanine, tryptophan, tyrosine, and valine and is thought to be up-regulated in such cells in order to support the high rate of protein synthesis required for various cell functions. The rat and human cDNAs encoding LAT1 have been identified and characterized^20^, and the LAT1 protein was shown to be localized in the basolateral membrane of polarized epithelia^21,22^. LAT1 was also found to be expressed at a high level in rat, mouse, and human pancreatic islets^23–25^ and to mediate the transport of large neutral amino acids with high affinity^21^ in a manner dependent on intracellular amino acid concentrations^26^. However, it has remained unknown whether amino acid influx is rate limiting for insulin production and secretion in pancreatic β cells.

Here we uncover a role for CD44v in regulation of insulin biosynthesis via LAT1. Depletion of CD44 in insulin-secreting cells resulted in increased amino acid influx mediated by LAT1 and increased insulin secretion. Furthermore, we found that the transport of amino acids by LAT1 is rate limiting for insulin biosynthesis. Our results suggest that the CD44v-LAT1-insulin axis is a potential target for the treatment of diabetes.

## Results

### CD44v expression is increased in islets of diabetic model mice

**Figure 1:**
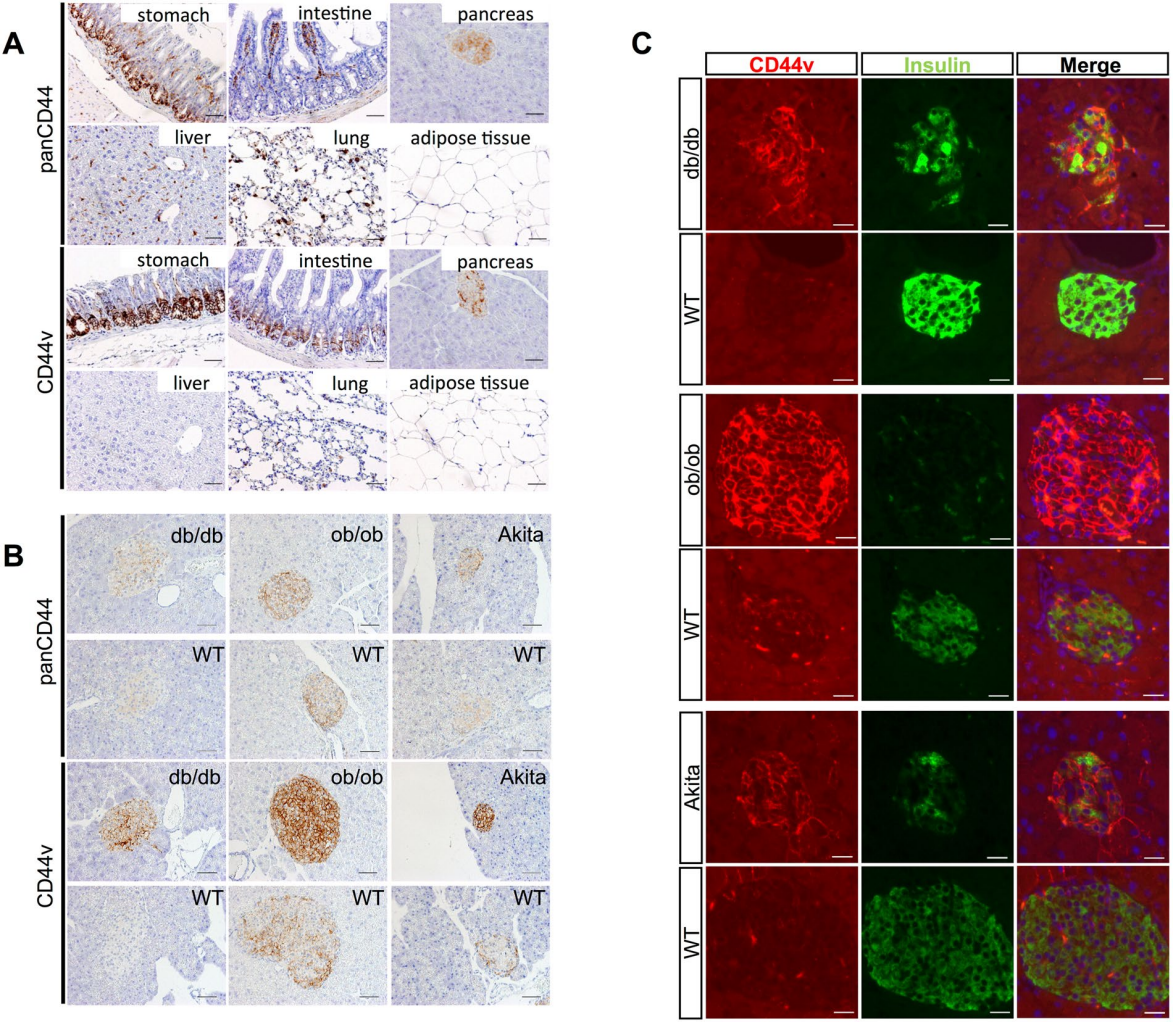
CD44v is highly expressed in pancreatic islets of diabetic mice. (**A**) Immunohistochemical staining of CD44 (panCD44) and CD44v in the stomach, intestine, pancreas, liver, lung, and adipose tissue of adult C57BL/6 mice. Scale bars, 50 µm. (**B**) Immunohistochemical staining of panCD44 and CD44v in pancreatic islets of adult db/db, ob/ob, and Akita diabetic mice and the corresponding WT animals. Scale bars, 50 µm. (**C**) Immunohistofluorescence staining of CD44v and insulin in pancreatic islets of adult db/db, ob/ob, and Akita diabetic mice and the corresponding WT animals. The fluorescence (blue) of nuclei counterstained with 4′,6-diamidino-2-phenylindole (DAPI) is shown in the merged images. Scale bars, 40 µm.

To investigate the possible role of CD44v in diabetes, we first examined its expression in normal mouse tissues. CD44 was previously detected in normal mouse tissues^27^, but we applied antibodies that recognize all forms of CD44 (panCD44) or CD44v specifically to immunohistochemical analysis. Such analysis revealed CD44v-positive cells in the pancreas, stomach, and intestine but not in the liver, lungs, or adipose tissue of C57BL/6 mice (Fig. 1A). Within the pancreas, the expression of CD44v appeared to be confined to the endocrine portion and absent from the exocrine component. To confirm this localization and to investigate the relevance of CD44v in pancreatic islets, we next examined its expression in mouse models of diabetes. Both db/db and ob/ob mice are characterized by obesity and overeating as a result of the loss of leptin signaling, and they develop hyperglycemia, impaired glucose tolerance, and hyperinsulinemia. Akita mice are a nonobese diabetic model with a point mutation in one allele of the insulin 2 gene (*Ins2*); they manifest high blood glucose (~500 mg/dl) and insulin levels, and insulin secretion from their isolated pancreatic islets in response to glucose stimulation is impaired^28–31^. Whereas CD44v was undetectable in exocrine pancreatic tissue of db/db, ob/ob, or Akita mice, it was expressed at a high level in the pancreatic islets of all these diabetic mice, most prominently and uniformly at cell membranes (Fig. 1B and Supplementary Fig. S1A–D). Immunohistofluorescence staining for both CD44v and insulin revealed that the abundance of CD44v was inversely related to that of insulin, with diabetic mice showing high CD44v and low insulin levels and wild-type (WT) mice showing high insulin and low CD44v levels (Fig. 1C). We confirmed that the abundance of CD44v had no change on other endocrine secretion in pancreatic β cells. The pattern and extent of glucagon immunoreactivity did not differ between diabetic and WT mice (Supplementary Fig. S1E,F). These results suggested that the abundance of CD44v was inversely related to insulin, but not to glucagon in pancreatic β cells.

### CD44 depletion increases insulin secretion and insulin content in Min6 cells

**Figure 2:**
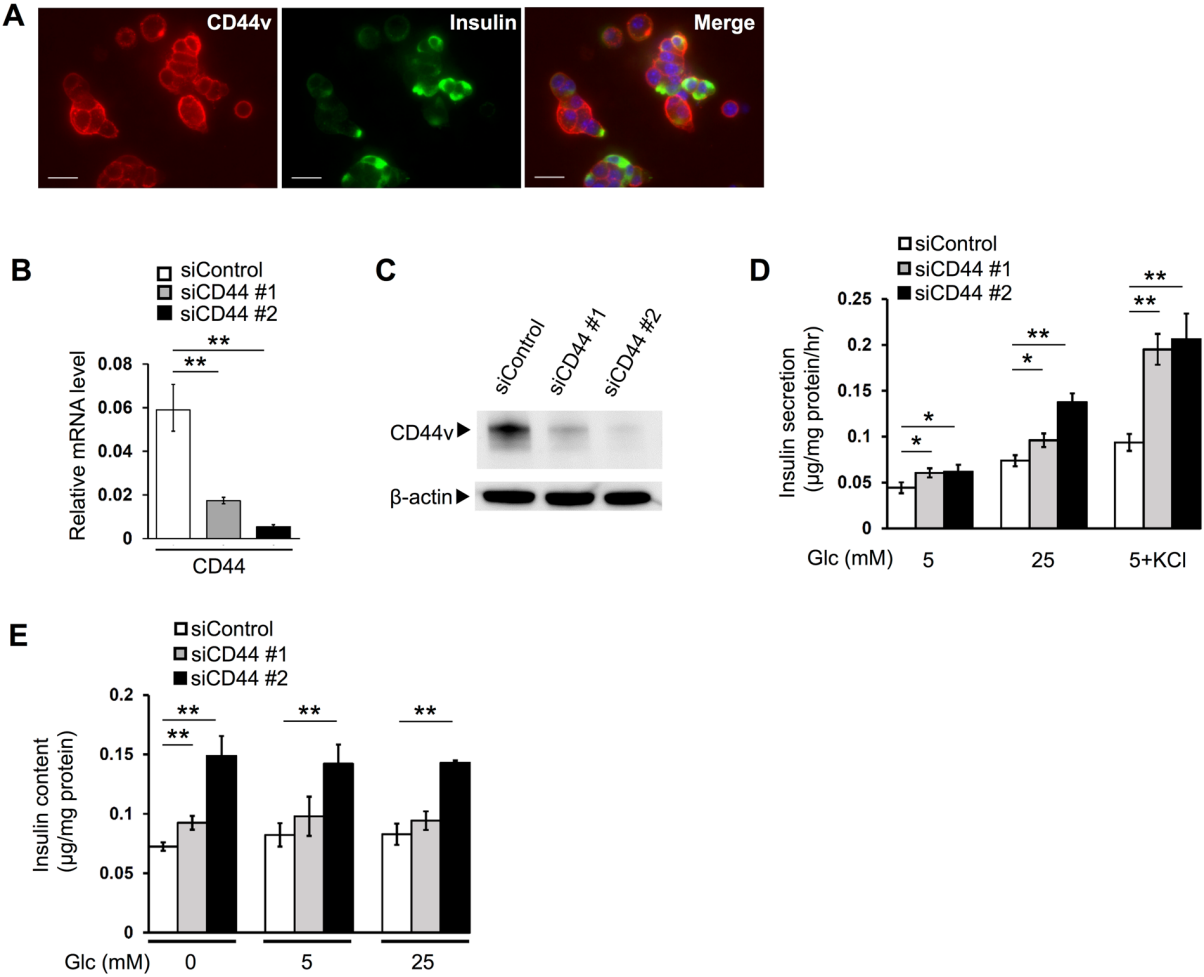
CD44 depletion increases insulin secretion and insulin content in Min6 cells. (**A**) Immunofluorescence staining for CD44v and insulin in Min6 cells. The fluorescence (blue) of nuclei counterstained with DAPI is shown in the merged image. Scale bars, 50 µm. (**B**) RT and real-time PCR analysis of CD44 gene transcripts in Min6 cells transfected with control or CD44 (#1 or #2) siRNAs. (**C**) Immunoblot analysis of CD44v and β-actin (loading control) in Min6 cells transfected as in (**B**). (**D**) Min6 cells transfected as in (**B**) were incubated for 1 h in the presence of 5 or 25 mM glucose or of 5 mM glucose (Glc) plus 30 mM KCl, after which the amount of insulin released into the incubation medium was determined with an ELISA. (**E**) The insulin content of Min6 cells transfected as in (**B**) was determined with an ELISA before and after incubation for 1 h in the presence of 5 or 25 mM glucose. All quantitative data are means ± s.d. for three independent experiments. *P < 0.05, **P < 0.01 (unpaired Student’s *t* test).

To explore further the relation between CD44v and insulin, we studied the pancreatic β-cell line Min6, which was established from a murine insulinoma and is widely adopted as a model for functional insulin-secreting cells^32,33^. We first confirmed that CD44v is expressed and localized at the cell membrane in Min6 cells (Fig. 2A). We then examined the effects of CD44 depletion on insulin secretion and content in these cells. Reverse transcription (RT)–polymerase chain reaction (PCR) and immunoblot analyses confirmed that transfection of Min6 cells with small interfering RNAs (siRNAs) specific for CD44 mRNA reduced the abundance of CD44 gene transcripts (Fig. 2B) as well as that of CD44v protein (Fig. 2C), respectively, compared with those in cells transfected with a control siRNA, with the efficacy of CD44 siRNA #2 being greater than that of CD44 siRNA #1. An enzyme-linked immunosorbent assay (ELISA) revealed that CD44 depletion with CD44 siRNAs #1 and #2 in Min6 cells increased insulin secretion over 1 h by 36% and 39%, respectively, in the presence of 5 mM glucose, by 30% and 87% in the presence of 25 mM glucose, and by 109% and 120% under conditions of maximal stimulation in the presence of 30 mM KCl plus 5 mM glucose (Fig. 2D). Of note, the insulin content of Min6 cells was also increased by transfection with CD44 siRNA #1 or #2, both at the steady state and after glucose stimulation for 1 h (Fig. 2E). CD44 depletion had no effect on the amounts of insulin gene (*Ins1* or *Ins2*) transcripts in Min6 cells (Supplementary Fig. S2). Together, these results provided further support for an inverse relation between CD44v abundance and intracellular insulin content as well as insulin secretion in Min6 cells.

### CD44 depletion changes intracellular amino acid levels in Min6 cells

**Figure 3:**
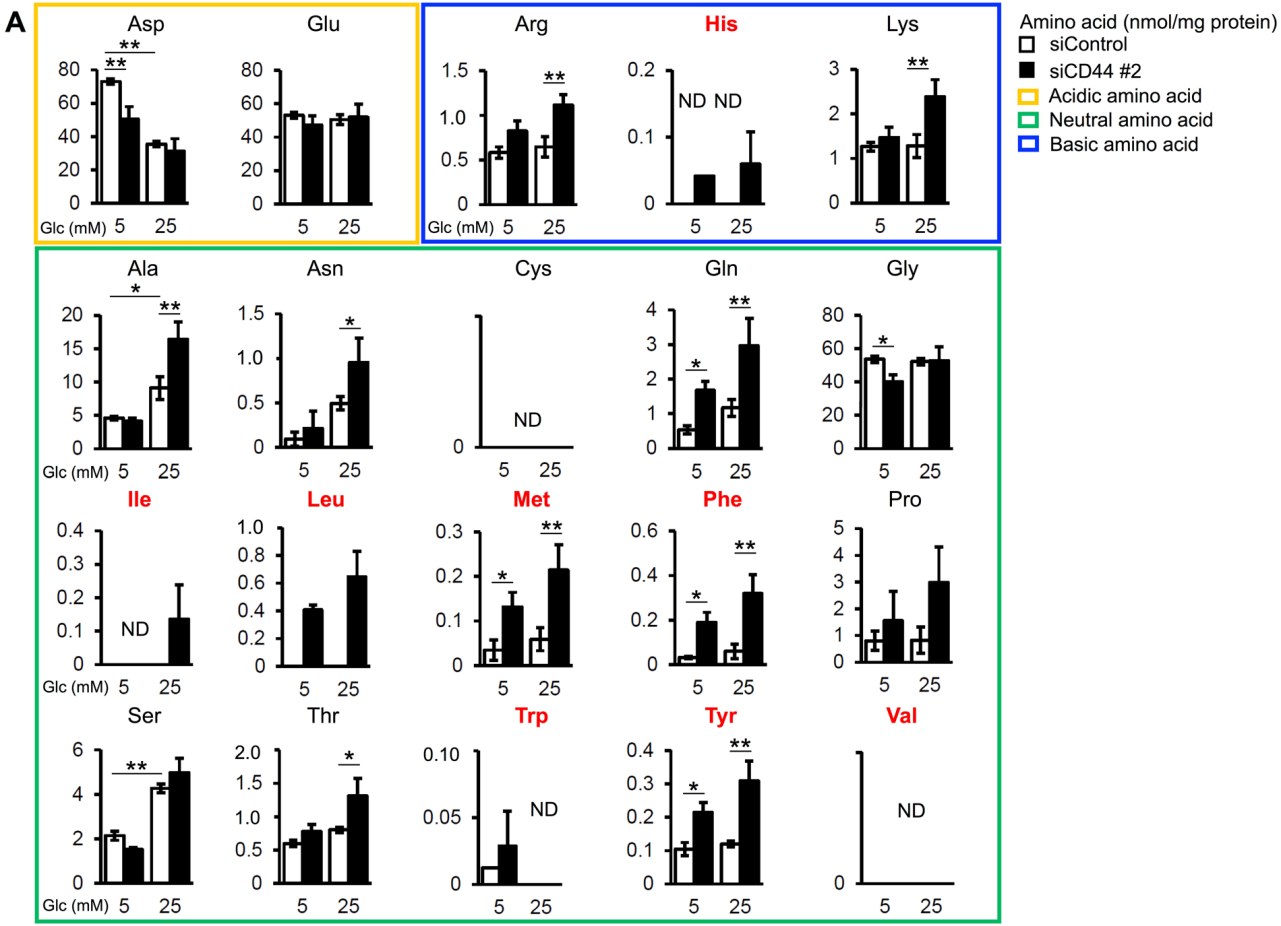
CD44 depletion influences intracellular levels of amino acids in Min6 cells. Min6 cells transfected with control or CD44 (#2) siRNAs and incubated in the presence of 5 or 25 mM glucose for 1 h were subjected to metabolome analysis by CE-MS. The amounts of amino acids (nmol/mg protein) were determined. Amino acids are shown classified according to their acidic (yellow frame), neutral (green frame), or basic (blue frame) nature. The abbreviations for amino acids transported into cells by LAT1 are shown in red. ND, not detected. Data are means ± s.d. for three independent experiments. *P < 0.05, **P < 0.01 (Tukey’s HSD and F test).

Given that the insulin content of CD44-depleted cells remained high despite a marked increase in insulin secretion and no change in the level of insulin gene transcripts, we next examined whether the effects of CD44 depletion might be related to insulin biosynthesis. Metabolome analysis by capillary electrophoresis and mass spectrometry (CE-MS) revealed that CD44 depletion resulted in a significant increase in the intracellular levels of several amino acids in Min6 cells (Fig. 3). Focusing on amino acids whose abundance was increased by both CD44 depletion and glucose stimulation, we found that most of these amino acids are transported into cells by the L-type amino acid transporter (LAT) system. Among them, leucine, methionine, phenylalanine, and tyrosine combined make up more than 19%, 30%, 19%, and 42% of insulin A, B, and C chains as well as the insulin signal peptide, respectively (Supplementary Fig. S3A). It should be noted that depletion of CD44 induced a significant decrease in levels of aspartic acid (Asp) which is not transferred into cells by LAT1 system. These results suggested that CD44v depletion in Min6 cells might promote the transport of amino acids by LAT1 and thereby increase the intracellular abundance of amino acids present in insulin and up-regulate insulin biosynthesis (Supplementary Fig. S3B).

### LAT1 inhibition reduces insulin secretion and insulin content in Min6 cells

**Figure 4:**
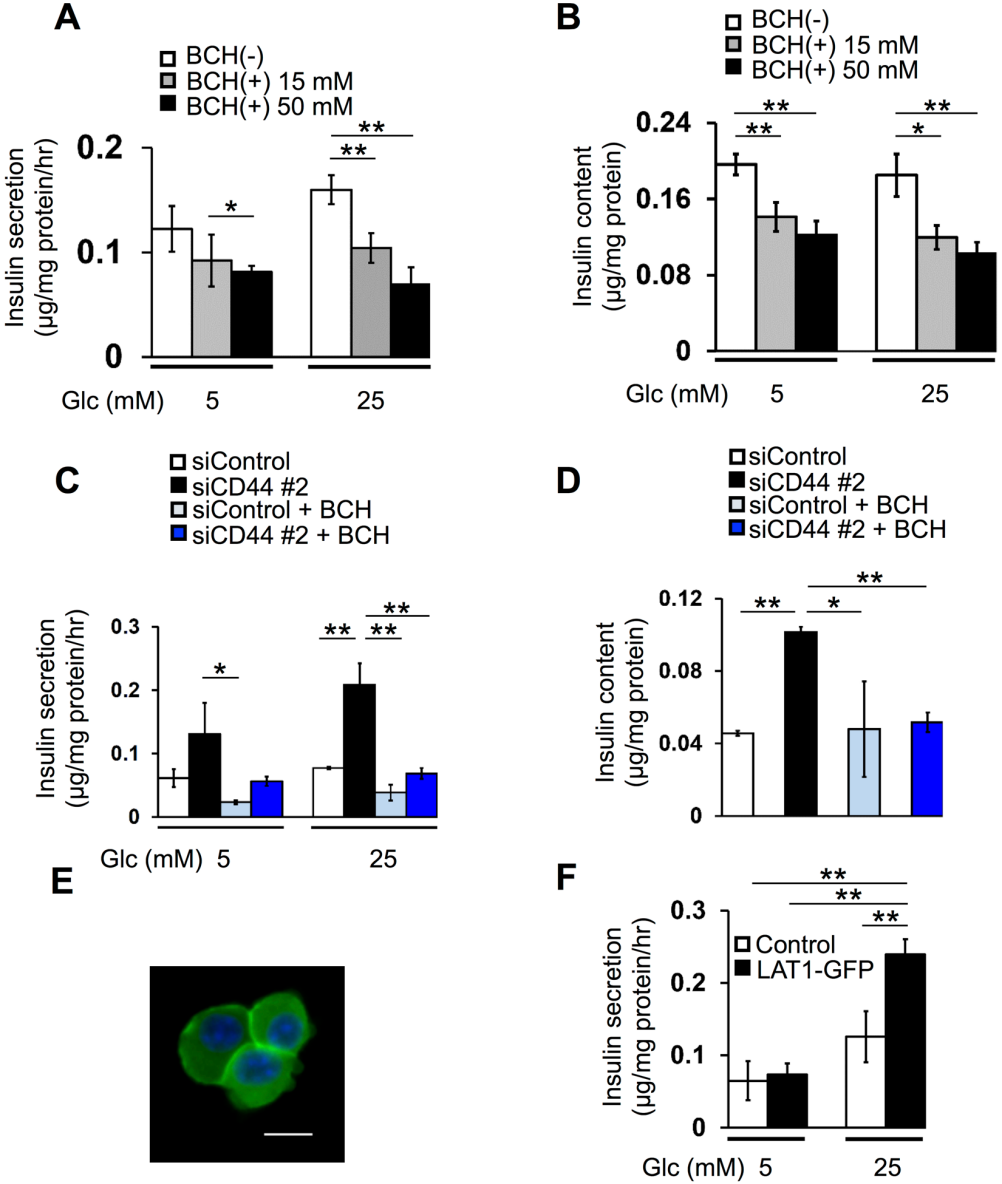
Amino acid transport by LAT1 supports insulin biosynthesis and secretion in Min6 cells. (**A**) Min6 cells that had been exposed to 0, 15, or 50 mM BCH for 24 h were incubated in the additional presence of 5 or 25 mM glucose for 1 h, after which the amount of insulin released into the incubation medium was determined. (**B**) Insulin content of Min6 cells treated as in (**A**). (**C**) Min6 cells that had been transfected with CD44 (#2) or control siRNAs and then exposed (or not) to 50 mM BCH for 24 h were incubated for 1 h in the additional presence of glucose at 5 or 25 mM for assay of insulin secretion. (**D**) Insulin content of Min6 cells that had been transfected with CD44 (#2) or control siRNAs and then incubated in the absence or presence of 50 mM BCH for 24 h. (**E**) Fluorescence microscopy of Min6 cells expressing LAT1-GFP. Nuclei were stained with DAPI (blue). Scale bar, 20 µm. (**F**) Min6 cells expressing LAT1-GFP or infected with the corresponding empty virus (Control) were incubated for 1 h in the presence of glucose at 5 or 25 mM for assay of insulin secretion. All quantitative data are means ± s.d. for three independent experiments. *P < 0.05, **P < 0.01 (unpaired Student’s *t* test).

To investigate the role of amino acid influx in insulin secretion, we first examined the effect of the LAT inhibitor 2-aminobicyclo-(2,2,1)-heptane-2-carboxylic acid (BCH)^20,21,34^ in Min6 cells. BCH attenuated insulin secretion over 1 h in a concentration-dependent manner in the presence of 5 or 25 mM glucose (Fig. 4A). Importantly, the insulin content of the cells after glucose stimulation for 1 h was also significantly reduced by treatment with BCH (Fig. 4B). Moreover, BCH treatment prevented the positive effects of CD44 depletion on both insulin secretion and intracellular insulin abundance (Fig. 4C,D). As was the case with siRNA-mediated CD44 knockdown (Supplementary Fig. S2), BCH treatment had no effect on the amounts of *Ins1* or *Ins2* transcripts in Min6 cells (Supplementary Fig. S4). Together, these results thus indicated that CD44 depletion increases the intracellular abundance of amino acids required for insulin biosynthesis and thereby promotes insulin production in a manner dependent on active transport through the LAT system.

We next infected Min6 cells with a retrovirus for a green fluorescent protein (GFP) fusion of LAT1 in order to clarify whether LAT1 itself increases insulin secretion by mediating the influx of amino acids. Fluorescence imaging confirmed expression of GFP in part at the plasma membrane (Fig. 4E). Expression of LAT1-GFP significantly increased insulin secretion over 1 h in the presence of 25 mM glucose (Fig. 4F). These results suggested that LAT1 is required for insulin secretion. Collectively, those data thus allow us to speculate that insulin secretion is regulated by LAT1-mediated amino acid transport and that CD44v inhibits insulin secretion by attenuating this function of LAT1.

### Expression of CD44v is inversely related to that of LAT1 in Min6 cells and mouse islets

**Figure 5:**
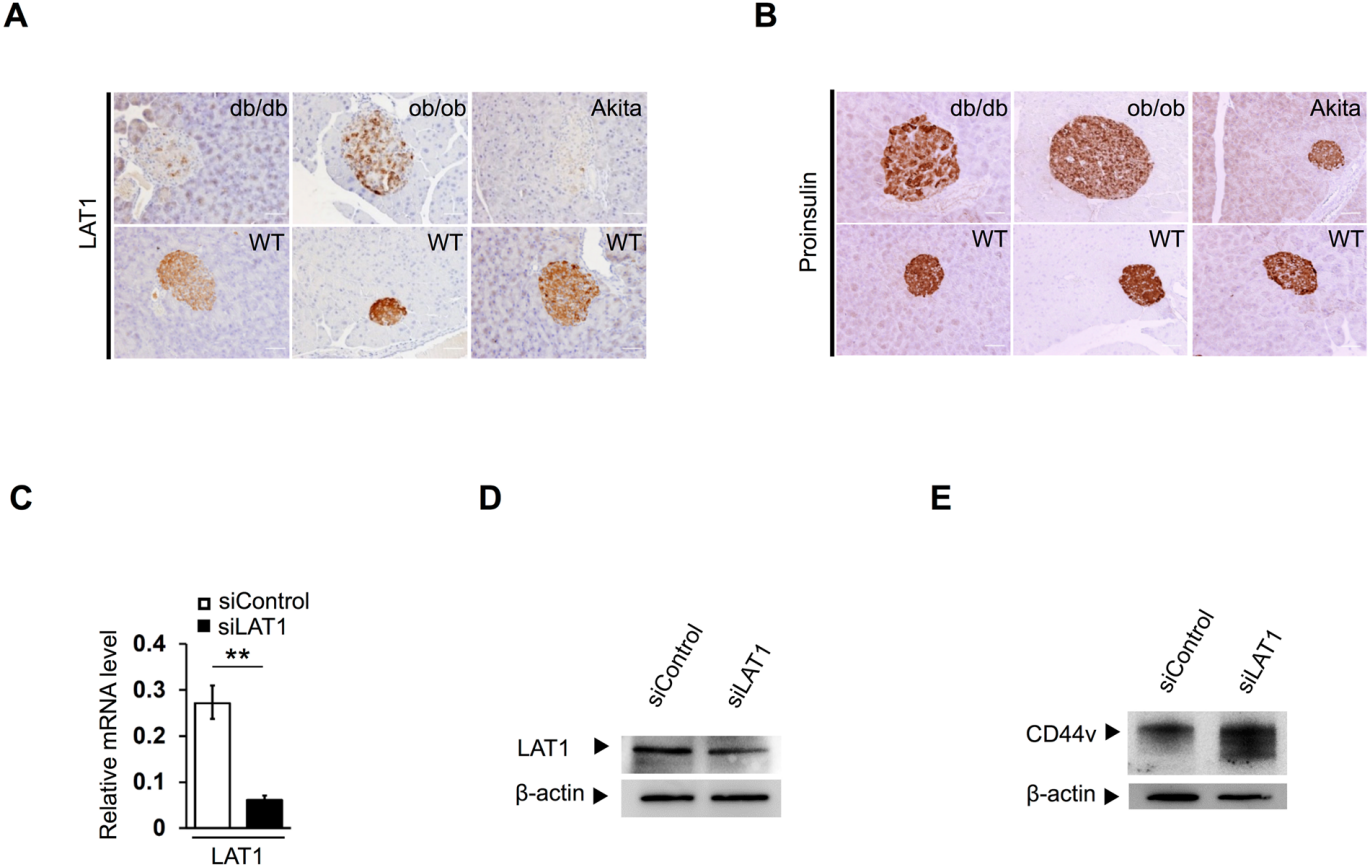
Relation between LAT1 and proinsulin expression in mouse islets and between CD44v and LAT1 expression in Min6 cells. (**A**,**B**) Immunohistochemical staining for LAT1 (**A**) and proinsulin (**B**) in pancreatic islets of db/db, ob/ob, and Akita diabetic mice as well as of their WT counterparts. Scale bars, 40 µm. (**C**) RT and real-time PCR analysis of LAT1 mRNA in Min6 cells transfected with control or LAT1 siRNAs. Data are means ± s.d. for three independent experiments. **P < 0.01 (unpaired Student’s *t* test). (**D**) Immunoblot analysis of LAT1 and β-actin in Min6 cells transfected as in (**C**). (**E**) Immunoblot analysis of CD44v in Min6 cells transfected as in (**C**).

To investigate the relation between CD44v and LAT1 in mouse pancreatic islets, we examined the expression of LAT1 in islets of normal and diabetic mice. Immunohistochemistry revealed that the abundance of LAT1 in pancreatic islets was lower in diabetic model mice than in their WT counterparts (Fig. 5A and Supplementary Fig. S5A,B), with this expression pattern thus being opposite to that of CD44v (Fig. 1B and Supplementary Fig. S1A–D). To examine the role of LAT1 in insulin biosynthesis *in vivo*, we also determined intracellular proinsulin levels in islets. The abundance of proinsulin was also slightly lower in the islets of all diabetic mice than in those of their WT counterparts (Fig. 5B and Supplementary Fig. S5C,D). The expression levels of both proinsulin and LAT1 thus tended to be lower in islets with a higher level of CD44v expression and vice versa. We next examined the effect of LAT1 depletion on CD44v expression in Min6 cells. RT and real-time PCR analysis and immunoblot analyses confirmed that transfection of Min6 cells with small interfering RNAs (siRNAs) specific for LAT1 mRNA reduced the abundance of LAT1 mRNA (Fig. 5C) as well as that of LAT1 protein (Fig. 5D), respectively, compared with that apparent in cells transfected with a control siRNA. Immunoblot analysis revealed that siRNA-mediated LAT1 depletion resulted in up-regulation of CD44v expression (Fig. 5E). These results thus provided further support for an inverse relation between the expression level of CD44v and those of LAT1 and intracellular insulin.

## Discussion

We have here shown that CD44v regulates insulin secretion in mouse pancreatic β cells and that the expression level of CD44v is inversely related to intracellular insulin and amino acid levels. We also found that the expression levels of CD44v and the neutral amino acid transporter LAT1 are inversely related in mouse pancreatic islets.

The primary purpose of our research has been to clarify the expression pattern and functional roles of CD44v in normal mouse tissues. Analysis of the expression of CD44 in various tissues of C57BL/6 mice with antibodies to both panCD44 and CD44v revealed that immunoreactivity with both types of antibodies in the pancreas was restricted to islet cells. Moreover, the level of CD44v expression was markedly increased in the islets of diabetic model (db/db/, ob/ob, and Akita) mice, all of which manifest a reduced level of insulin secretion. These results thus suggested that CD44v might contribute to the negative regulation of insulin secretion. A link between CD44 expression and autoimmune insulitis has been described previously^12^, but a role for CD44 in insulin secretion has not. To clarify whether insulin secretion and intracellular insulin content in pancreatic β cells might be regulated directly by CD44v, we studied the mouse Min6 cell line, which mimics pancreatic β-cell function.

We found that depletion of all isoforms of CD44 by RNA interference in Min6 cells resulted in enhancement of insulin secretion, suggesting that CD44v inhibits insulin secretion in mouse β cells. Furthermore, CD44 depletion resulted in an increase in intracellular insulin content in Min6 cells. These findings contrast with previous results showing an inverse relation between insulin secretion and insulin content^35^. Our data thus suggested that CD44 depletion might promote insulin biosynthesis. The observation that depletion of CD44 had no effect on the abundance of insulin gene (*Ins1* or *Ins2*) transcripts in Min6 cells further suggested that such promotion of insulin biosynthesis might be mediated at a posttranscriptional level. Metabolome analysis revealed that CD44 depletion in Min6 cells increased the intracellular levels of several amino acids including leucine, methionine, phenylalanine, and tyrosine as well as the intracellular insulin content. The relation between amino acids and insulin is complex^36^. Neutral and branched-chain amino acids are both building blocks of insulin^37^ and regulators of insulin secretion^38,39^. Leucine is the most effective activator of mechanistic target of rapamycin complex 1 (mTORC1)^40–43^, which plays a key role in the regulation of protein biosynthesis. LAT1 is thought to mediate cellular uptake of leucine leading to mTORC1 activation^44–46^. Amino acids for protein synthesis are also provided by uptake from the extracellular environment mediated by transporters at the plasma membrane^47–49^. An increase in LAT1 activity might thus have contributed to the increased intracellular abundance of amino acids and insulin in Min6 cells depleted of CD44.

We next investigated the effects of LAT1 inhibition on insulin secretion and biosynthesis. Inhibition of the LAT system with BCH resulted in down-regulation of both insulin content and secretion in Min6 cells, consistent with the positive relation between intracellular amino acid levels and insulin abundance^50^. Metabolome analysis revealed that glucose stimulation was accompanied by significant increases in the intracellular levels of 2 of the 15 neutral amino acids in Min6 cells transfected with a control siRNA. The concentration-dependent inhibitory effect of BCH on insulin secretion thus indicated that intracellular amino acid abundance is rate limiting for this process. Consistent with this notion, we found that overexpression of LAT1 enhanced glucose-induced insulin secretion. Moreover, inhibition of LAT1 by BCH prevented the increase in insulin content and secretion induced by CD44 depletion in Min6 cells, suggesting that CD44v inhibits insulin biosynthesis and secretion by restraining LAT1-mediated amino acid uptake.

The relation between increased CD44v expression and reduced insulin secretion in three different types of diabetic model mice, with each model reflecting a different type of islet dysfunction^28–31^, implicates CD44v in the pathophysiology of diabetes. The expression level of CD44v was also inversely related to that of LAT1 in islets of normal and diabetic mice. Moreover, LAT1 depletion increased CD44v expression in Min6 cells, consistent with the notion that the CD44v-LAT1 interaction influences insulin biosynthesis. Although the LAT system does not mediate net vectorial amino acid transfer, it complements the action of unidirectional transporters localized in the same membrane^26^. Certain amino acids that are efflux substrates of LAT1-4F2hc may thus be exchanged for other amino acids that are conponents of insulin.

Cell growth is regulated by mTOR, the overactivation of which induces cancer progression^51,52^. The molecular mechanism by which CD44 depletion promotes the influx of amino acids via LAT1 remains to be determined. In addition, given that CD44v interacts with xCT and that both xCT and LAT1 are alternative light chains that form transporter complexes with the CD98 heavy chain, the possibility that xCT may modulate the regulation of LAT1 by CD44v warrants further investigation.

Our results thus implicate CD44v in the regulation of insulin biosynthesis and secretion via LAT1, and they suggest that the insulin biosynthetic capacity of β cells is dependent on LAT1-mediated amino acid transport. Our findings thus identify the CD44v-LAT1-insulin axis as a potential target for therapeutic intervention in diabetes.

## Methods

### Mice

All animal experiments were approved by the Animal Care and Use Committee of Keio University. Eight- to 16-week-old male mice (db/db-C57BLKS, WT-C57BLKS, ob/ob-C57BL/6 J, Akita-C57BL/6 J, and WT-C57BL/6 J) were obtained from Charles River (Yokohama, Japan), group-housed, and provided with water and a standard laboratory diet ad libitum. All animals were bred and maintained in the animal facility at Keio University according to institutional guidelines.

### Cell culture

Min6 cells were kindly provided by Jun-ichi Miyazaki^32^ (Osaka University) and were cultured under 5% CO_2_ at 37 °C in High-Glucose (25 mM) Dulbecco’s modified Eagle’s medium–F12 (Sigma-Aldrich, St. Louis, MO) supplemented with 10% fetal bovine serum, 1% penicillin-streptomycin, and 72 μM β-mercaptoethanol^53^.

### Immunostaining

Mouse tissues were fixed in 4% paraformaldehyde, embedded in paraffin, and sectioned at a thickness of 4 µm. The sections were exposed for 1 h to phosphate-buffered saline (PBS) containing 3% bovine serum albumin, incubated overnight at 4 °C with primary antibodies, washed three times with PBS, and then incubated for 1 h at room temperature with secondary antibodies. Primary antibodies included those to insulin (Abcam, Cambridge, MA), to proinsulin (R&D Systems, Minneapolis, MN), to LAT1 (Transgenic, Kumamoto, Japan), to panCD44 (clone IM7; BD Pharmingen, Franklin Lakes, NJ), to CD44v (Link Genomics, Tokyo, Japan), and to glucagon (Cell Signaling Technology, Danvers, MA). For immunohistochemistry, immune complexes were detected with Histofine Simple Stain (Nichirei, Tokyo, Japan). Images were captured with a microscope (Olympus), and staining was quantified with Image J software (NIH, Bethesda, MD). For immunohistofluorescence staining, immune complexes were detected with Alexa Fluor 594– or Alexa Fluor 488–conjugated secondary antibodies (Molecular Probes, Eugene, OR). Nuclei were counterstained with 4′,6-diamidino-2-phenylindole (DAPI).

For immunocytofluorescence staining, cells were fixed with 4% paraformaldehyde, permeabilized for 10 min with PBS containing 0.1% Triton X-100, exposed to PBS containing 3% bovine serum albumin for 1 h, and then incubated for 2 h at room temperature with primary antibodies. The cells were then washed three times with PBS and incubated for 1 h at room temperature with Alexa Fluor–labeled secondary antibodies. Nuclei were counterstained with Hoechst 33342. Immunofluorescence images were acquired with a BZ9000 inverted fluorescence microscope (Keyence, Osaka, Japan) and digitally processed with Keyence Analysis Software.

### Immunoblot analysis

Cells were lysed in radioimmunoprecipitation assay (RIPA) buffer (Sigma-Aldrich), the lysates were fractionated by SDS-polyacrylamide gel electrophoresis, and the separated proteins were transferred to a polyvinylidene difluoride membrane. Immunoblot analysis was performed with antibodies to β-actin (clone C-4; Santa Cruz Biotechnology, Santa Cruz, CA), CD44v (Link Genomics), and to LAT1 (Santa Cruz Biotechnology).

### RT and real-time PCR analysis

Total RNA was isolated from Min6 cells with the use of an RNeasy kit (Qiagen, Hilden, German) and was subjected to RT with a Prime Script RT reagent kit (Takara Clontech, Shiga, Japan) followed by real-time PCR analysis with SYBR Green gene expression assays (Qiagen). Primers (sense and antisense, respectively) for mouse mRNAs included those for β-actin (5′-CTGGCTCCTAGCACCATGAAGAT-3′ and 5′-GGTGGACAGTGAGGCCAGGAT-3′), panCD44 (5′-TCCGAATTAGCTGGACACTC-3′ and 5′-CCACACCTTCTCCTACTATTGA-3′), LAT1 (5′-TCTTCCTCATTGCCGTGTCATTC-3′ and 5′-GCTTGTTCTTCCACCAGACACC-3′), INS1 (5′-GAAGCGTGGCATTGTGGAT-3′ and 5′-TGGGCCTTAGTTGCAGTAGTTCT-3′), and INS2 (5′-GCTTCTTCTACACACCCATGTC-3′ and 5′-AGCACTGATCTACAATGCCAC-3′). The abundance of target mRNAs was normalized by that of β-actin mRNA.

### RNA interference

CD44 siRNAs were designed to specifically knockdown all splicing variants of the mouse CD44 gene. The siRNA sequences (sense and antisense, respectively) were as follows: CD44 siRNA #1, 5′-UUCCUUCGAUGGACCGGUUtt-3′ and 5′-AACCGGUCCAUCGAAGGAAtt-3′; CD44 siRNA #2, 5′-GUCUCAGGAAAUGGUGCAUtt-3′ and 5′-AUGCACCAUUUCCUGAGACtt-3′; and scrambled control siRNA, 5′-UUCUCCGAACGUGUCACGUtt-3′ and 5′-ACGUGACACGUUCGGAGAAtt-3′. The sequences for a LAT1 siRNA were 5′- GUCAUGUCCUGGAUCAUUCtt-3′ and 5′-GAAUGAUCCAGGACAUGACac-3′.

Min6 cells (3 × 10^5^ per well of six-well plates) were transfected for 48 h with 100 pmol of siRNA with the use of the Lipofectamine RNA iMAX reagent (Invitrogen, Carlsbad, CA).

### Expression vectors and cell transfection

Mouse LAT1 cDNA was amplified by PCR from total cDNA derived from the mouse breast cancer cell line 4T1 (American Type Culture Collection, Rockville, MD) and was cloned upstream of enhanced GFP cDNA into the pMXs-IP vector for expression of a LAT1-GFP fusion protein. The modified pMXs-IP vector together with pVSV-G was introduced by transfection into GP2-293 cells with the use of the FuGene reagent (Promega, Madison, WI). The retroviruses released into the culture supernatant were then harvested for infection of Min6 cells. Infected cells were selected with puromycin (2 µg/ml).

### BCH treatment

BCH^20,34,54^ was obtained from Sigma-Aldrich. Min6 cells (3 × 10^5^ per well of six-well plates) were cultured for 24 h and then exposed to various concentrations of BCH in culture medium for 24 h before analysis of insulin secretion or insulin content.

### Measurement of insulin secretion and insulin content

Min6 cells (3 × 10^5^ per well) were cultured in six-well plates for 48 h, washed with PBS, and then incubated at 37 °C first for 60 min in Krebs-Ringer bicarbonate buffer (140 mM NaCl, 3.6 mM KCl, 0.5 mM NaH_2_PO_4_, 0.5 mM MgSO_4_, 1.5 mM CaCl_2_, 10 mM Hepes, and 2 mM NaHCO_3_ at pH 7.4) and then for 60 min in the same solution supplemented with 5 or 25 mM glucose. Insulin released into the incubation buffer or present in cell lysates was then measured with the use of a mouse insulin ELISA kit (Morinaga, Tokyo, Japan) and was normalized by the amount of total protein in each well.

### Analysis of metabolites by CE-MS

Cells transfected with control or CD44 siRNAs were plated in 10-cm dishes and incubated with 5 or 25 mM glucose for 1 h, after which metabolites were extracted according to the Human Metabolome Technologies (HMT, Tsuruoka, Japan) metabolite extraction method for adherent cells^55^. Metabolite concentrations were measured with the use of an Agilent Capillary Electrophoresis System (HMT)^56,57^ and were normalized by the amount of cellular protein.

### Statistical analysis

Quantitative data are presented as means ± s.d. and were analyzed with the unpaired Student’s *t* test as performed with Excel 2013 software (Microsoft, Redmond, WA), with the exception that metabolome data were analyzed with Tukey’s HSD and F test. A *P* value of <0.05 was considered statistically significant.

### Data availability

The datasets generated during and/or analysed during the current study are available from the corresponding author on reasonable request.

## Electronic supplementary material

Supplement Information
