# Author Correction: CD44 variant inhibits insulin secretion in pancreatic β cells by attenuating LAT1-mediated amino acid uptake

**DOI:** 10.1038/s41598-020-63111-7

**Published:** 2020-04-03

**Authors:** Nana Kobayashi, Shogo Okazaki, Oltea Sampetrean, Junichiro Irie, Hiroshi Itoh, Hideyuki Saya

**Affiliations:** 1 Division of Gene Regulation, Institute for Advanced Medical Research, Keio University School of Medicine, Tokyo, 160-8582 Japan; 2 Division of Endocrinology, Metabolism, and Nephrology, Department of Internal Medicine, Keio University School of Medicine, Tokyo, 160-8582 Japan

Correction to: *Scientific Reports* 10.1038/s41598-018-20973-2, published online 12 February 2018

This Article contains errors. The information in this Article is incomplete. The clone for the CD44v antibody is RM1. It is now available from Cosmo Bio USA.

