# Supplementary material for: CD44 variant inhibits insulin secretion in pancreatic β cells by attenuating LAT1-mediated amino acid uptake
Source: Sci Rep. 2018 Feb 12;8:2785. doi: 10.1038/s41598-018-20973-2 (PMC5809395; doi:10.1038/s41598-018-20973-2)
Supplement: Supplementary file 1 — Supplement Information [file 41598_2018_20973_MOESM1_ESM.docx]

**Supplementary figures for**

**CD44 variant inhibits insulin secretion in pancreatic β cells**

**by attenuating LAT1-mediated amino acid uptake**

**Nana Kobayashi^1, 2^, Shogo Okazaki^1^, Oltea Sampetrean^1^, Junichiro Irie^2^, Hiroshi Itoh^2^ & Hideyuki Saya^1^***

^1^Division of Gene Regulation, Institute for Advanced Medical Research, Keio University School of Medicine, Tokyo 160-8582, Japan.

^2^Division of Endocrinology, Metabolism, and Nephrology, Department of Internal Medicine, Keio University School of Medicine, Tokyo 160-8582, Japan.

*Correspondence and requests for materials should be addressed to H.S. (email: hsaya@a5.keio.jp).


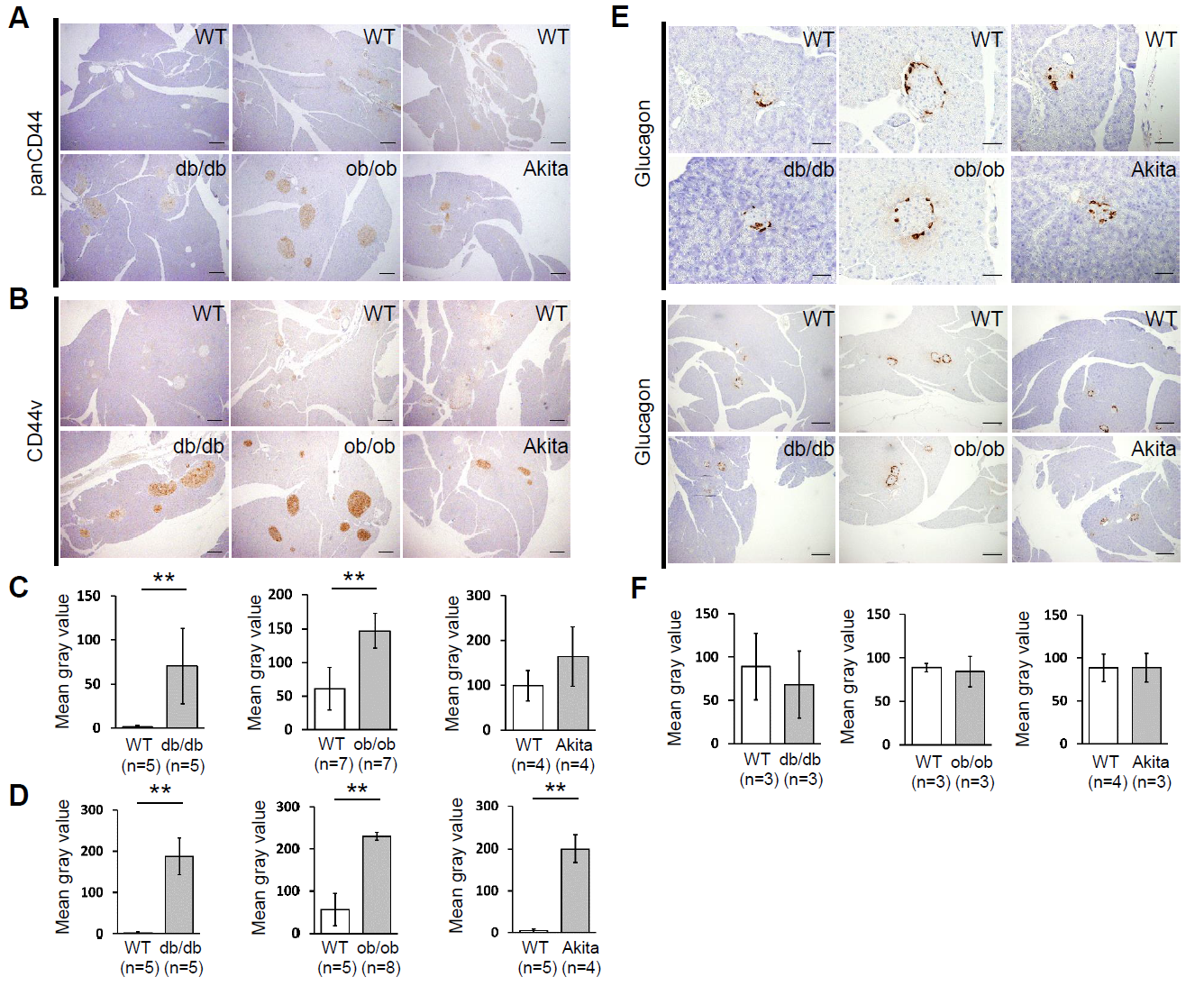


**Supplementary Figure S1. Expression of CD44v in endocrine glands, but not in exocrine glands, of the pancreas in WT and diabetic model mice.** (**A, B**) Pancreatic sections from adult db/db, ob/ob, and Akita diabetic mice as well as from the corresponding WT animals were subjected to immunohistochemical analysis with antibodies to panCD44 (**A**) and to CD44v (**B**). Scale bars, 200 µm. (**C, D**) Quantification of panCD44 (**C**) and CD44v (**D**) immunostaining in islets of adult db/db, ob/ob, and Akita diabetic mice as well as of the corresponding WT animals. (**E**) Pancreatic sections from adult db/db, ob/ob, and Akita diabetic mice as well as from the corresponding WT animals were subjected to immunohistochemical analysis with antibodies to glucagon. Scale bars, 50 µm (upper panels) and 200 µm (lower panels). (**F**) Quantification of glucagon immunostaining in sections similar to those in (**E**). All quantitative data are means ± s.d. for 3-8 islets. n: number of islets measured the staining intensity. ***P* < 0.01 (unpaired Student’s *t* test).


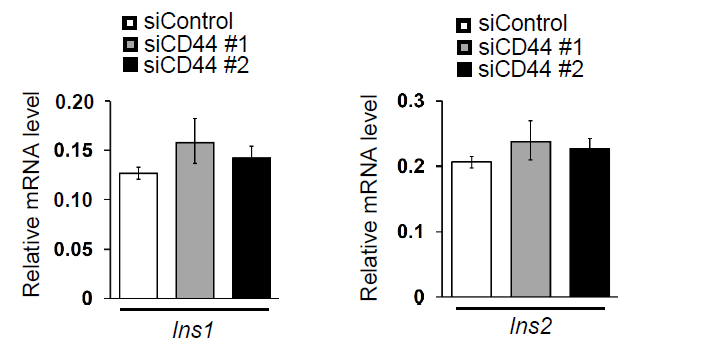


**Supplementary Figure S2. Depletion of CD44 in Min6 cells does not affect the abundance of insulin gene transcripts.** The abundance of *Ins1* and *Ins2* transcripts in Min6 cells transfected with control or CD44 siRNAs was determined by RT and real-time PCR analysis. Data are means ± s.d. for three independent experiments.


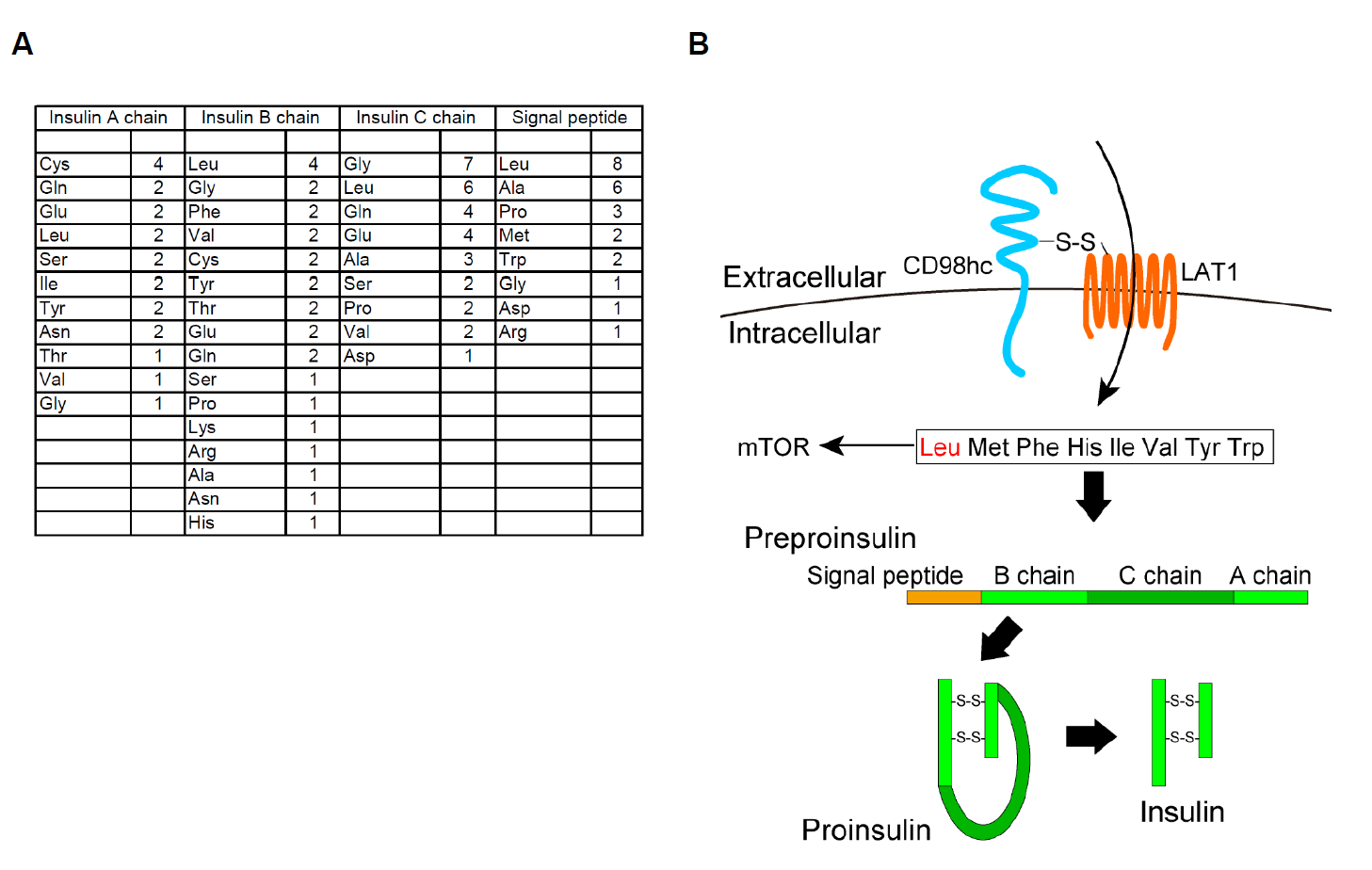


**Supplementary Figure S3. Role of LAT1 in regulation of intracellular amino acid levels and insulin secretion in Min6 cells.** (**A**) Amino acid composition of the various portions of mouse preproinsulin. (**B**) Model for the role of LAT1 in regulation of intracellular amino acid levels and insulin secretion in Min6 cells.


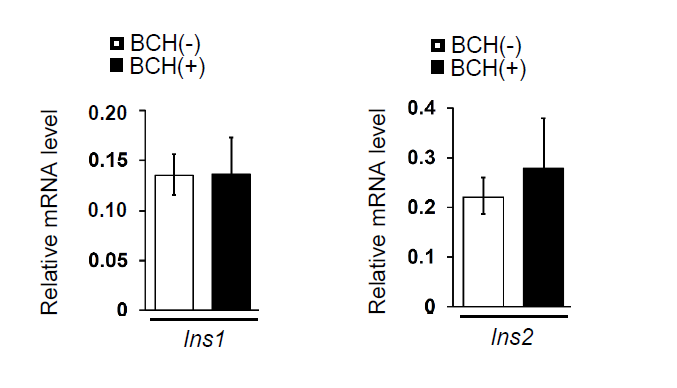


**Supplementary Figure S4. BCH treatment does not affect the abundance of insulin gene transcripts in Min6 cells.** The abundance of *Ins1* and *Ins2* transcripts in Min6 cells incubated for 24 h in the absence or presence of 50 mM BCH was determined by RT and real-time PCR analysis. Data are means ± s.d. for three independent experiments.


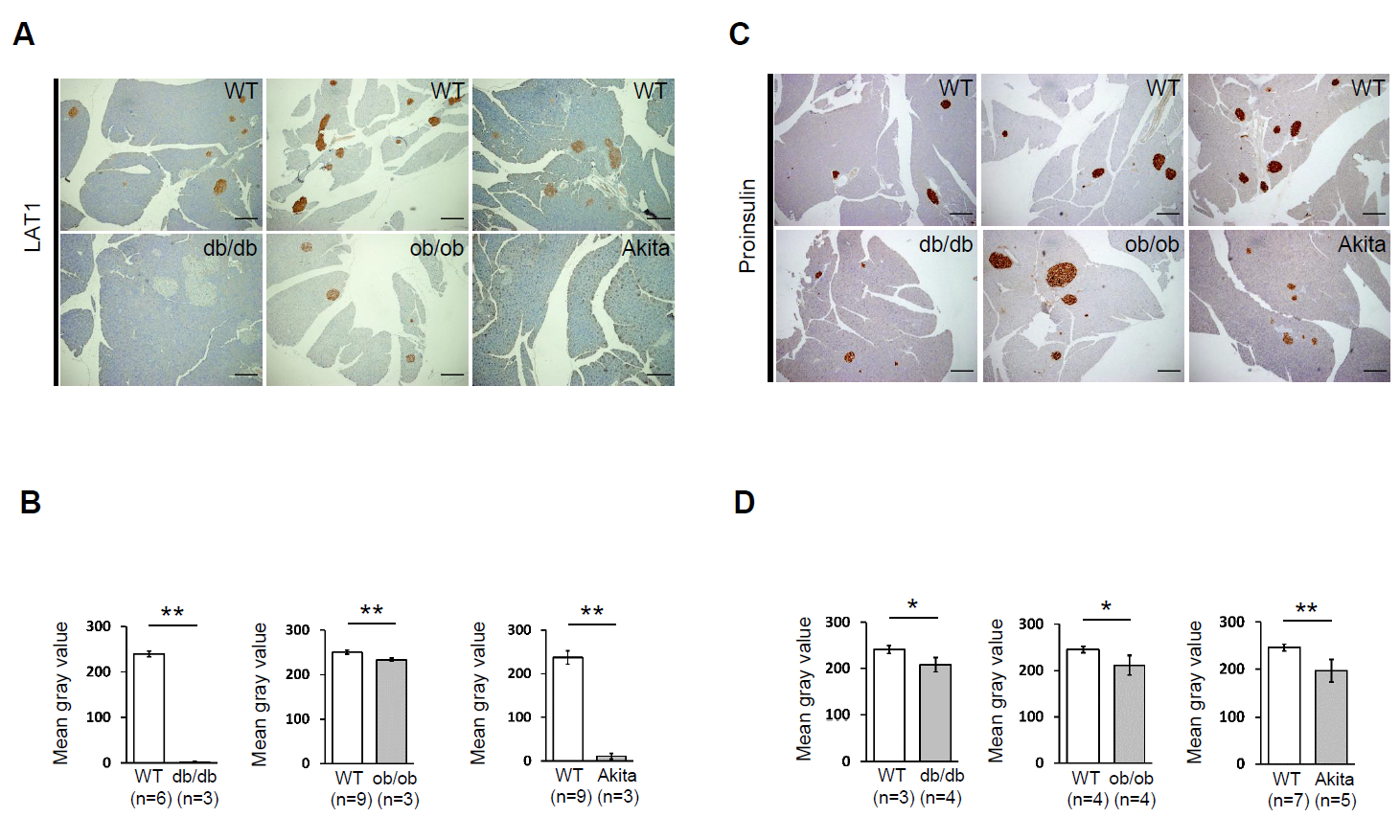


**Supplementary Figure S5. Expression of LAT1 and proinsulin in islets of WT and diabetic model mice.** Pancreatic sections from adult db/db, ob/ob, and Akita diabetic mice as well as from the corresponding WT animals were subjected to immunohistochemical analysis with antibodies to LAT1 (**A**) and to proinsulin (**C**), and LAT1 (**B**) and proinsulin (**D**) immunostaining was then quantified. Scale bars, 200 µm. Quantitative data are means ± s.d. for 3-9 islets. n: number of islets measured the staining intensity. **P* < 0.05, ***P* < 0.01 (unpaired Student’s *t* test).
